# Analysis of Damping Characteristics of Magnetorheological Damper under Impact Load

**DOI:** 10.3390/ma15124161

**Published:** 2022-06-12

**Authors:** Min Sun, Xiangdong Li, Zhou Zhou, Qibin Zhu, Bing Liu, Xu Chen, Jiong Wang, Guang Zhang, Shibo Cai

**Affiliations:** 1 Special Equipment Safety Supervision Inspection Institute of Jiangsu Province, Nanjing 210002, China; jstjxz2022@163.com (M.S.); jstjxz2023@163.com (Z.Z.); workin0516@163.com (Q.Z.); workin202215@163.com (B.L.); ccube1984@163.com (X.C.); 2 School of Mechanical Engineering, Nanjing University of Science and Technology, Nanjing 210094, China; wjiongz@njust.edu.cn (J.W.); guangzhang@zjut.edu.cn (G.Z.); 3 College of Mechanical Engineering, Zhejiang University of Technology, Hangzhou 310014, China; ccc@zjut.edu.cn; 4 XGM Corporation Limited, Taizhou 317100, China; 5 Beijing Advanced Innovation Center for Intelligent Robots and Systems, Beijing Institute of Technology, Beijing 100081, China

**Keywords:** magnetorheological gel, impact load, damping coefficient, Couette shear flow, Poiseuille pressure flow

## Abstract

Compared to magnetorheological fluid, magnetorheological gel has better anti-settling performance and stability. Therefore, magnetorheological gel is suitable for devices that can meet operational requirements in all aspects after long-term storage, such as the anti-recoil application of weapons. To study this in-depth, the mechanism of the influence of magnetorheological gel micro-magnetic-mechanical properties on the macro-output damping mechanics of the damper, a parallel plate model of the mixed flow mode composed of Couette shear flow and Poiseuille pressure flow was established. The theoretical analysis was of the output damping of the damper. Finally, the controllability of the damper under impact load employed magnetorheological gel was preliminarily analyzed. The results indicate that the damping coefficient of the damper increases with the increase of dynamic viscosity $\eta_{B}$ of the magnetorheological gel, piston effective cross-sectional area $A_{P}$, magnetic pole L, and Bingham coefficient Bi. Magnetorheological damper has controllability under impact load and can reach a wide controllable range under the condition under small magnetic field ranging from 0 mT to 131 mT.

## 1. Introduction

Magnetorheological gel (MRG) is a kind of MR smart material, which is composed micro/sub-micro soft magnetic material particles, i.e., pure iron, carbonyl, and nickel, uniformly dispersed in the non-magnetic matrix such as polyurethane and silicone in a certain proportion. A multi-state material with the following rheological properties, it has extreme fluidity without a magnetic field, and the mechanical properties of the material are uniform and isotropic. Once a magnetic field is applied, the microstructure changes rapidly and continuously. The particles follow the direction of the magnetic field arranged in a chain column or net shape, the mechanical behaviors of the material indicate obvious anisotropy, have a certain yield strength, and immediately return to the original state after the magnetic field is evacuated [1,2,3,4,5,6]. The matrix is a polymer material with a three-dimensional network structure. Therefore, MRG has better anti-settling and anti-aggregation properties than magnetorheological fluid (MRF). In addition, since the polyurethane matrix has a higher viscosity than silicone oil, the sealing structure of the device using MRG as the medium is simple. Based on the above properties, and because of its low energy consumption, wide adaptable temperature range, low pollution, and easy control [7,8], it can be widely used in various suspension damping systems, optical spherical lens polishing, and tactile sensing [9,10,11,12].

For example, the fluid damping channel area of the anti-recoil device of the traditional artillery is usually a constant or a function of the buffer stroke. It is difficult to adjust damping in real time according to the external shooting conditions, and it cannot meet the technical performance requirement of artillery proposed by the variability of the application environment in modern warfare [13,14,15,16]. Based on the good electromechanical coupling characteristics of magnetorheological smart materials, great results are achieved in anti-vibration control, and it also provides the possibility for the shock buffer control design of electromechanical systems under complex conditions [17]. Wang et al. proposed the application of magnetorheological damper to the recoil of artillery [18], which established a dynamic model of magnetorheological damper and discussed the application of magnetorheological damper in application on the anti-recoil device of the artillery. Subsequently, the team conducted research on the dynamic characteristics of the magnetorheological damper under the impact of the artillery barrel force [19]. Since 2003, Wang J and Hou B have achieved fruitful results in the structural design and control of the magnetorheological damper used in the anti-recoil device of the artillery [20,21,22]. Li et al. designed a magnetorheological damper-based recoil for a certain type of artillery and conducted dynamic tests under five different currents to verify the feasibility of the damper for recoil control [23]. Zhu established a dynamic model and an electromagnetic model for a certain type of artillery, designed a magnetorheological recoil system, and proposed PID and fuzzy control algorithms [24]. Ahmadian designed a magneto-rheological damper suitable for high-speed artillery recoil and established a dynamic model of the recoil process, confirming the possibility of magnetorheological damper applied to artillery recoil [25]. Bajkowski et al. studied the cushioning performance of the magnetorheological buffer used in the recoil damping system of the AKMS carbine [26,27]. Harinder and Norman proposed a multi-objective optimization problem based on minimizing recoil load and maximizing firepower. The mechanical model predicts the recoil force of the damper by evaluating the pressure [28]. Deepak et al. designed a magnetorheological damper for artillery recoil and compared it to a traditional passive brake-based artillery damping device [29].

The above works have conducted focus on the possibility, mechanical characteristics, and damping output performance of the dampers developed by the magnetorheological fluid as the power transmission medium for artillery recoil, reflecting the feasibility of applying the dampers to weapon systems. Further analysis of the controllable micro-mechanism was conducted. Micro-rheological properties based on magnetorheological materials have a great important influence on the macro-output damping performance of the damper. Therefore, to obtain the mechanism of how the micro-damped channel rheological properties regulate the macroscopic output damping of the damper and to further understand the essential relationship between them, this paper establishes the process of recoil Parallel plate model of the damping channel under the impact load, which employs the resultant force in the barrel during the recoiling process of the artillery as the analysis background. Aiming at the model in the mixed flow mode, the relationship between the MRG micro-mechanical performance and the macro-damping output of the damper was theoretically analyzed. Finally, the MRG mechanical parameters were used to conduct a preliminary analysis of the controllability of the damper in the anti-recoil period of the gun.

## 2. Sample Preparation and Model Parameter Identification

### 2.1. Preparation

The synthesis of modified epoxy silicone resin was divided into three steps [30,31]. In the first step, the materials presented in Table 1 underwent polycondensation to generate hydroxy or ethoxy-containing silicone oligomers (intermediates). Secondly, under the action of a catalyst, the organic silicon intermediate obtained in the first step and the epoxy resin were condensed in a solvent to form a modified epoxy silicone resin. Finally, carbonyl iron powder (Model: JCF2-2, Jilin Jien Nickel Industry Co., Ltd., China, with an average diameter of 1 μm) was used as magnetic particles and mixed with the silicone resin matrix evenly.

**Figure 1:**
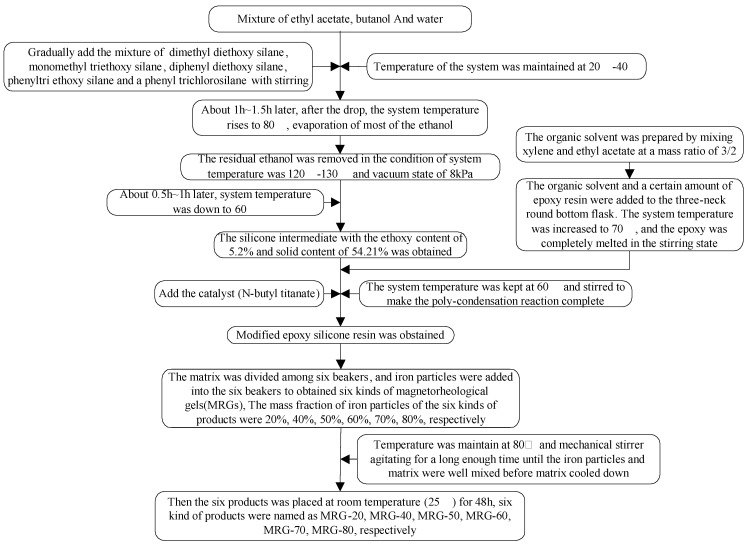
Preparation process of silicone-based MRG.

The schematic diagram of the preparation process of silicone resin-based MRG is presented in Figure 1. The preparation process was divided into three steps: preparation of organosilicon oligomer (intermediate), preparation of modified epoxy silicone resin (finished product), and preparation of silicone resin-based magnetorheological adhesive.

**Figure 2:**
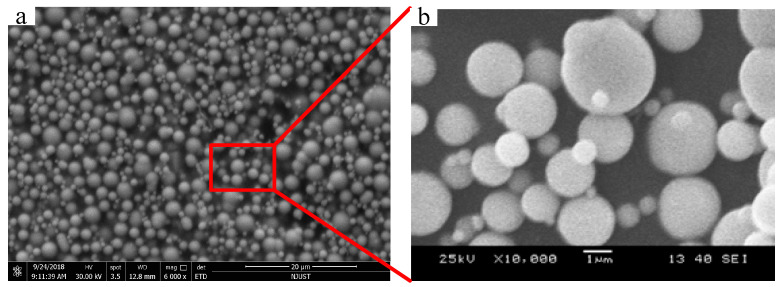
Scanning electron microscope of MRG-70 (**a**) magnification = 10,000, (**b**) magnification =200,000.

According to the mass fraction of carbonyl iron powder, the MRG was named MRG-70. From the reference [32], it can be indicated that the MRG-70 MRG-70 has the best magnetorheological effect. When the magnetic induction intensity of MRG-70 is 480 mT, the magnetorheological effect reaches the maximum value of 188.8%, which can meet the vibration control requirements of magnetorheological devices. The prepared MRG-70 was scanned by an electron microscope to observe the uniformity of the internal carbon-based iron powder distribution. The result is presented in Figure 2. It can be observed from Figure 2 that the diameter of the carbonyl iron powder (sphere) is between 200 nm–3 μm, and the carbonyl iron powder in the self-made MRG-70 is evenly distributed in the silicone resin matrix.

### 2.2. Bingham Model Parameter Identification

**Figure 3:**
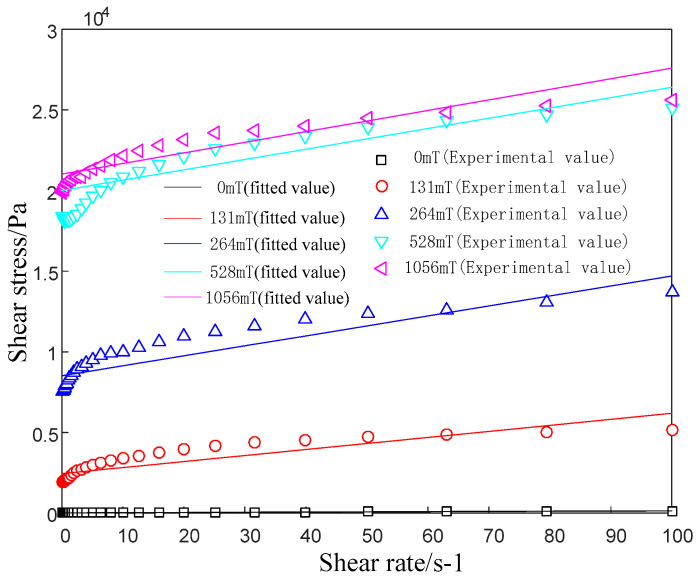
Flow curves at different magnetic induction intensity for MRG-70.

A commercial rheometer (model: Anton Paar MCR physica 302) was used to measure the flow curve of MRG-70 at a magnetic induction intensity of B = [0,131,264,528,1056] mT at room temperature (25 °C), and the range of shear rate changes. For 0–100 s^−1^, the result is presented in Figure 3. It is worth mentioning that under the action of the magnetic field, the internal particles of the material form chains in the direction of the magnetic field. Once the shear force is applied, the particle chain is destroyed, but reorganization occurs under the action of the magnetic field, so it is a process of continuous destruction and reorganization. Since the matrix of the material is a polymer material, the polymers are entangled with each other before shearing. Once shearing occurs, the polymer chains are unwrapped and arranged relatively orderly along the shearing direction.

It can be observed from Figure 3 that for different magnetic fields, the relationship between the shear stress and the shear rate approximately satisfies a linear change after yields. The shear yield stress (the corresponding shear stress when the shear rate is close to 0) increases with the increase of the magnetic field. Based on the above analysis, MRG-70 is a non-Newtonian fluid, and its constitutive properties can be described by a simple Bingham model [32,33,34]:

(1) $$\{\begin{array}{c} \tau =\tau_{B}+\eta_{B}\dot{\gamma }\;\;\;\;\;\;\;\tau >\tau_{B}\;\\ \dot{\gamma }=0\;\;\;\;\;\;\;\;\;\;\;\;\;\;\tau \leq \tau_{B} \end{array}\;\;\;\;$$

where, τ is shear stress, $\tau_{B}$ is magneto-induced shear yield stress, $\eta_{B}\frac{dU_{Bm}\left(y\right)}{dy}$ is shear stress caused by flow, $\eta_{B}$ is the dynamic viscosity of the fluid and is related to the magnetic field, and $\dot{\gamma }$ is shear rate. Figure 3 also indicates the fitting results of the Bingham model. The model parameter identification under different magnetic fields is presented in Table 2.

## 3. Dynamic Analysis for MR Damper System (Take the Anti-Recoil System of Artillery for Example)

**Figure 4:**
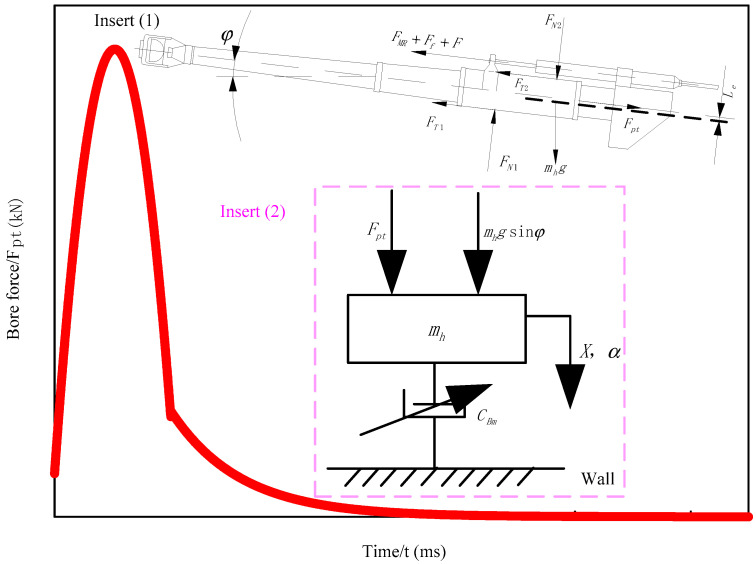
The curve of time of bore resultant force of a certain type of fixed artillery.

When the artillery is launched, the primary driving force for the recoil movement of the artillery is the resultant force of the barrel under the action of the gunpowder gas. This force is short, about tens of milliseconds, but the effect on the body of the gun is very complicated. To facilitate the analysis, the resultant force of the barrel is divided into two stages: the barrel period (research object) and the after-effect period of gunpowder. Figure 4 presents the change curve of the resultant force of a certain type of fixed artillery barrel with time [35]. The resultant bore force $F_{pt1}$ in the boring period along with time can be expressed using the following formula [36,37,38]:

(2) $$F_{pt1}=\frac{1}{\varepsilon }\left(1+\frac{1}{2}\frac{\omega }{m}\right)Ap\approx Ap$$

where *ω* is the charge quality, *m* is projectile quality, *ε* is calculated the coefficient for the minor work, *A* is the ballistic cross-sectional area, $p=sin\left(\frac{\pi }{\gamma }t\right)$ is average pressure in the bore, and γ is average pressure coefficient in the barrel.

It can be observed from the curve in Figure 4 that the barrel period of this type of artillery has the characteristics of short action time, violent changes, and high peaks when fired. Therefore, an anti-recoil device is required to dissipate the impact energy regularly during recoil. Figure 4 insert (1) is a schematic diagram of the force for the self-designed magnetorheological buffer applied to the recoil.

As presented in Figure 4 insert (1), the main power received by the recoil during the launch is the resultant force $F_{pt1}$ of the barrel and the gravity $m_{h}g$ of the recoil, which act on the axis of the barrel and the center of mass of the recoil, respectively. In addition, the restraining reaction force includes magnetorheological buffer resistance $F_{MR}$, recoil force $F_{f}$, and friction force F of their sealing device, in addition to normal force $F_{N1}$, $F_{N2}$ and corresponding friction force $F_{T1},F_{T2}$ of the cradle rail. The total friction force on the cradle rail is:

(3) $$F_{T}=F_{T1}+F_{T2}=f\left(F_{N1}+F_{N2}\right)$$

where f is the coefficient of friction of cradle rail.

According to the D’Alembert principle of the mass point, the main force, the restraining force, and the inertial force acting on the recoiling part form a balanced force system, so the differential equation of the recoil motion of the artillery can be expressed as:

(4) $$F_{pt}+m_{h}gsin\varphi -m_{h}\frac{d^{2}X}{dt^{2}}-F_{MR}-F_{f}-F-F_{T}=0$$

where X is recoil stroke and φ is gun height. Because the artillery recoil process is very complicated, the coupling of the magnetorheological damper adds to the complexity of the analysis system. Therefore, in the magnetorheological recoil model, the recoil process of the artillery during recoil is not considered [22]. Figure 4 insert (2) presents the single-degree-of-freedom dynamic model for the magnetorheological recoil motion of artillery.

Since having in-depth research on the resultant force of the bore during the recoil of the artillery, the theory of the resultant force of the bore during the aftereffect period is still based on various assumptions and calculations based on empirical formulas in practical applications. Therefore, the following primarily focuses on the analysis of the damping characteristics of the magnetorheological damper during the recoil movement of the gun. According to Figure 4 insert (2), the dynamics differential equation of magnetorheological artillery anti-recoil can be described as follows [39]:

(5) $$m_{h}\frac{d^{2}X}{dt^{2}}=F_{pt}+m_{h}gsin\varphi -F_{MR}=F_{pt}+m_{h}gsin\varphi -C_{Bm}\frac{dX}{dt}$$

During the barrel period, the recoil of the artillery began to move from stillness, so the boundary conditions are as follows:

(6) $$\{\begin{array}{c} X\left(0\right)=0\\ X^{\prime }\left(0\right)=0 \end{array}$$

where $C_{Bm}$ is the effective damping coefficient of the magnetorheological damper, which is related to the strength of the magnetic field.

Under the effect of the change regulation of the resultant force of the barrel in the barrel period presented in Figure 4, Equation (5) is integrated and substituted into the boundary conditions to obtain the analytical equation of the movement displacement with time during the recoil period as follows:

(7) $$X=-\frac{1000\times 116}{C_{Bm}}\left[\frac{t^{3}}{3}-2\frac{m_{h}}{C_{Bm}}\left(\frac{t^{2}}{2}-\frac{m_{h}}{C_{Bm}}t\right)\right]+\frac{1000\times 600}{C_{Bm}}\left(\frac{t^{2}}{2}-\frac{m_{h}}{C_{Bm}}t\right)+\frac{1000+m_{h}gsin\varphi }{C_{Bm}}t\;\;\;+\frac{m_{h}}{C_{Bm}}\left(\frac{232\times 1000m_{h}^{2}}{C_{Bm}{}^{3}}+\frac{1000\times 600m_{h}}{C_{Bm}{}^{2}}-\frac{1000+m_{h}gsin\varphi }{C_{Bm}}\right)\;\;\;\;-\frac{m_{h}}{C_{Bm}}\left(\frac{232\times 1000m_{h}^{2}}{C_{Bm}{}^{3}}+\frac{1000\times 600m_{h}}{C_{Bm}{}^{2}}-\frac{1000+m_{h}gsin\varphi }{C_{Bm}}\right)e^{-\frac{C_{Bm}}{m_{h}}t}$$

For a specific type of artillery in a specific firing state, its mass $m_{h}$ and high and low firing angles *φ* are known. The resultant force $F_{pt}$ of the barrel during the barrel period has been obtained previously. The only unknown element is the effective damping coefficient $C_{Bm}$ of the magnetorheological damper. In addition, due to the large recoil force, very short action time, and dramatic changes during the recoil period of the artillery, the following part mainly analyzes the magnetorheological damping coefficient $C_{Bm}$ under the condition of ∆p≫v (under a strong magnetic field or under large yield stress).

## 4. Analysis of the Magnetorheological Damping Coefficient $C_{Bm}$

For establishing the parallel plate model inside the damping channel under the working condition of the damper, we focused on the case of the parallel plate model working in the mixed flow mode (the actual working state of the damper) composed of Couette shear flow and Poiseuille pressure flow. The damping effect of the damper under the Bingham constitutive model is analyzed, and the damping coefficient $C_{Bm}$ of the damper is obtained. Table 3 presents the boundary conditions of the Bingham fluid in the mixed-mode, and the representative meanings of each match will be explained in the following analysis.

**Figure 5:**
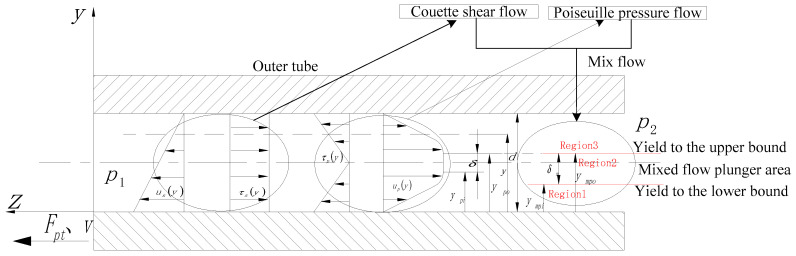
A parallel plate model of Bingham constitutive fluid flow in the damping channel.

We took the left end of the piston as the origin and the direction of piston movement as the *z*-axis to establish a rectangular coordinate system as displayed in Figure 5. The damper is moved by the external force $F_{pt}$ at the speed v, and the fluid flow in the damping channel is very complicated. This model describes the continuous development of quasi-steady flow, including Couette shear flow and Poiseuille pressure flow (hereinafter referred to as shear flow and pressure flow). It is specifically described as following in the mixed flow parallel plate model; shear flow is the movement of a pole plate (piston) to drive fluid movement in the absence of a pressure difference, and its movement direction is the same as that of the pole plate. Pressure flow is caused by the movement caused by the pressure difference between the inlet and outlet of the parallel plates. In the damper, the direction of movement is opposite to that of the piston. Therefore, the fluid flow in the damping channel of the damper is a mixed flow of shear flow and pressure flow. In addition, it is worth noting that in the actual damper, due to the movement of the piston, the fluid continues to flow from the extrusion cavity to the expansion cavity. Therefore, it can be considered that the fluid flow model of the damping channel is mainly pressure flow, while shear flow has a non-negligible effect on the characteristic area of its damping channel.

Figure 5 is a parallel plate model of Bingham constitutive fluid pressure flowing in a damping channel. The constitutive equation in this state can be described as follows [32]:

(8) $$\tau_{Bm}\left(y\right)=\tau_{B}+\eta_{B}\frac{dU_{Bm}\left(y\right)}{dy}$$

It can be observed from Figure 5 that under the mixed flow of the damping channel, the regional distribution of the plunger flow of the Bingham constitutive fluid is different from that of the pure pressure flow. The additional shear stress caused by the movement of the piston itself causes the flow to change in Region1, and then change the boundary conditions at $y_{mpi}$ (as presented in Table 4). The effect on the plunger flow in region2 can be described as follows: the shear stress caused by the piston movement is the applied basis of the pressure flow. Under the condition that the shear yield stress of MRG-70, the external pressure difference and the structural size of the damper does not change. The boundary conditions at the position of $y_{mpo}$ and $y_{mpo}$ do not change compared with pure pressure flow. Based on the above analysis, the flow situation of Region3 is consistent with the situation of pure pressure flow. The specific analysis is as follows:

Region1:

This area is the post-yield region. The flow velocity in this area under the parallel plate model is the vector sum of the flow velocity distribution of the damping channel under pure shear flow and pure pressure flow. It does not occur in the other directions since their flow velocity directions are all axial. The flow rate in this area can be described as follows:

(9) $$u_{Bm1}\left(y\right)=\frac{∆p}{2\eta_{B}L}y^{2}+C_{1}y+C_{2}+v\left(1-\frac{y}{d}\right)$$

where $C_{1},\;C_{2}$ is an undetermined coefficient, $d=R_{2}-R_{1}$, L is pole length, $∆p=p_{1}-p_{2}=-\frac{F_{pt}}{A_{p}}$, and $A_{p}$ is the effective working cross-sectional area of the piston. According to the boundary conditions listed in Table 4, the boundary conditions are substituted into Equation (9) to obtain the velocity distribution equation of the mixed flow of Bingham fluid in the damping channel:

(10) $$u_{Bm1}\left(y\right)=\frac{∆p}{2\eta_{B}L}y^{2}+\left(\frac{v}{d}-\frac{∆p}{\eta_{B}L}y_{mpi}\right)y+v\left(1-\frac{y}{d}\right)$$

Region2:

Since the form of external force and the geometric shape of the damper remain unchanged, the stress distribution between the parallel plates under the Bingham constitutive model is consistent with the Newtonian fluid:

(11) $$\tau_{Bm2}\left(y\right)=\frac{∆p}{L}y-\frac{∆pd}{2L}$$

Substituting the boundary conditions listed in Table 4 into Equation (11) and sorting out the y-axis direction width $\delta_{m}$ of the plunger flow before yielding:

(12) $$\delta_{m}=y_{mpo}-y_{mpi}=-\frac{\left(2\tau_{B}+\frac{\eta_{B}v}{d}\right)L}{∆p}=\frac{\left(2\tau_{B}+\frac{\eta_{B}v}{d}\right)L}{\left|∆p\right|}$$

Through the previous analysis, it was concluded that the upper yield boundary of the mixed flow plunger zone is consistent with the pressure flow, and the shear flow mainly affects the lower yield boundary position and the flow characteristics of Region1. Therefore, the boundary relationship of the plunger flow in the mixed flow is:

(13) $$d-\frac{\eta_{B}vL}{d\left|∆p\right|}=y_{mpo}-y_{mpi}$$

Simultaneous Equations (12) and (13) have:

(14) $$\{\begin{array}{c} y_{mpo}=\frac{1}{2}\left(\delta_{m}+d-\frac{\eta_{B}vL}{d\left|∆p\right|}\right)\\ y_{mpi}=\frac{1}{2}\left(d-\delta_{m}-\frac{\eta_{B}vL}{d\left|∆p\right|}\right) \end{array}$$

The dimensionless processing of Equation (14) gives:

(15) $$\{\begin{array}{c} y_{mpo}=\frac{d}{2}\left({\bar{\delta }}_{m}+1-\frac{\eta_{B}vL}{d^{2}\left|∆p\right|}\right)\\ y_{mpi}=\frac{d}{2}\left(1-{\bar{\delta }}_{m}-\frac{\eta_{B}vL}{d^{2}\left|∆p\right|}\right) \end{array}$$

where, ${\bar{\delta }}_{m}=\frac{\delta_{m}}{d}$ is dimensionless plunger flow width.

Region3:

According to Equation (9), the pressure-flow velocity distribution equation of Bingham fluid between parallel plates is separated as follows:

(16) $$u_{mp}\left(y\right)=\frac{∆p}{2\eta_{B}L}y^{2}+c_{1}y+c_{2}$$

Substituting the boundary conditions listed in Table 4 into Equation (16), the flow velocity distribution equation in Region3 in the parallel plate model of the mixed flow for the Bingham fluid in the damping channel is obtained:

(17) $$u_{mp3}\left(y\right)=\frac{∆p}{2\eta_{B}L}\left[y^{2}-d^{2}+2y_{mpo}\left(d-y\right)\right]$$

Substitute Equation (14) into Equations (10) and (17) to obtain the velocity equations of each region in the parallel plate model:

(18) $$\{\begin{array}{c} u_{Bm1}\left(y\right)=\frac{∆p}{2\eta_{B}L}y^{2}-\left[\frac{∆p}{2\eta_{B}L}\left(d-\delta_{m}\right)+\frac{v}{2d}\right]y+v\\ u_{Bm2}\left(y\right)=-\frac{∆p}{8\eta_{B}L}{\left(d-\delta_{m}-\frac{\eta_{B}vL}{d\left|∆p\right|}\right)}^{2}+v\\ u_{Bm3}\left(y\right)=\frac{∆p}{2\eta_{B}L}\left[y^{2}-d^{2}+\left(\delta_{m}+d-\frac{\eta_{B}vL}{d\left|∆p\right|}\right)\left(d-y\right)\right] \end{array}$$

Substituting Equation (18) into Equation (8) to obtain the shear stress distribution equation in each region of the parallel plate model:

(19) $$\{\begin{array}{c} \tau_{Bm1}\left(y\right)=\tau_{B}+\frac{\eta_{B}v}{d}+\frac{∆p}{L}y-\frac{∆p}{2L}\left(d-\delta_{m}\right)+\frac{\eta_{B}v}{2d}\\ \tau_{Bm2}\left(y\right)=\frac{∆p}{2L}\left(2y-d\right)\\ \tau_{Bm3}\left(y\right)=-\tau_{B}+\frac{∆p}{2L}\left[2y-\left(\delta_{m}+d-\frac{\eta_{B}vL}{d\left|∆p\right|}\right)\right] \end{array}$$

The shear stress caused by the viscous flow of the fluid under the Bingham model is consistent with the Newtonian fluid. The difference is that the Bingham model adds shear yield stress caused by the magnetic field. For this reason, the Bingham coefficient Bi is introduced, and the shear stress of the Newtonian fluid between the parallel plates is $\tau_{Bs}\left(y\right)=-\frac{\mu v}{d}$. Therefore, the one-dimensional quasi-steady-state shear stress between the parallel plates of the Bingham model is obtained:

(20) $$\tau_{Bm}\left(y\right)=-\frac{\eta_{B}v}{d}\left(1+Bi\right)$$

Comparing Equations (8) and (20), the expression of Bingham coefficient Bi is obtained as follows:

(21) $$Bi=\frac{\tau_{B}d}{\eta_{B}v}$$

According to Equation (12), the expression of shear yield stress is obtained:

(22) $$\tau_{B}=\frac{{\bar{\delta }}_{m}F_{pt}d}{2A_{p}L}-\frac{\eta_{B}v}{2d}$$

Substituting Equation (22) into Equation (21), the expression for the velocity of the piston movement caused by the bore force in the parallel plate model is obtained:

(23) $$v=\frac{{\bar{\delta }}_{m}F_{pt}d^{2}}{\eta_{B}A_{p}L\left(2Bi+1\right)}$$

The piston moves under the action of the bore force $F_{pt}$, and the damping force received is $C_{Bm}v$, where $C_{Bm}$ is the damping coefficient of the damper in the mixed flow mode of the Bingham fluid. From the perspective of dynamics, the relationship between force and velocity is obtained as:

(24) $$F_{pt}=C_{Bm}v$$

Substituting Equation (23) into Equation (24) to obtain the effective damping coefficient expression of the damper in the mixed flow mode:

(25) $$C_{Bm}=\frac{\eta_{B}A_{p}L\left(2Bi+1\right)}{{\bar{\delta }}_{m}d^{2}}$$

The flow rate of each area in the parallel plate model of the damping channel in the mixed flow mode is as follows:

(26) $$\left\{ \begin{array}{c} Q_{Bm1}=W\int_{0}^{y_{mpi}}u_{Bm1}\left(y\right)dy=-\frac{∆pWd^{3}}{96\eta_{B}L}{\left(1-{\bar{\delta }}_{m}+\frac{\eta_{B}vL}{∆pd^{2}}\right)}^{3}+\frac{vWd}{2}\left(1-{\bar{\delta }}_{m}+\frac{\eta_{B}vL}{∆pd^{2}}\right)\\ Q_{Bm2}=W\int_{y_{mpi}}^{y_{mpo}}u_{Bm2}\left(y\right)dy=-\frac{\bar{\delta }∆pWd^{3}}{8\eta_{B}L}{\left(1-{\bar{\delta }}_{m}+\frac{\eta_{B}vL}{∆pd^{2}}\right)}^{2}\\ Q_{Bm3}=W\int_{y_{mpo}}^{d}u_{Bm3}\left(y\right)dy=\frac{∆pWd^{3}}{2\eta_{B}L}\left[\frac{1}{12}{\left(1+{\bar{\delta }}_{m}+\frac{\eta_{B}vL}{∆pd^{2}}\right)}^{3}-\frac{1}{2}{\left(1+{\bar{\delta }}_{m}+\frac{\eta_{B}vL}{∆pd^{2}}\right)}^{2}+\left(1+{\bar{\delta }}_{m}+\frac{\eta_{B}vL}{∆pd^{2}}\right)-\frac{2}{3}\right] \end{array}\right.$$

where $W=2\pi R_{1}$, and the sum of the flow in the parallel plate model of the damping channel is $Q_{Bm}=Q_{Bm1}+Q_{Bm2}+Q_{Bm3}$. So:

(27) $$Q_{Bm}=-\frac{∆pWd^{3}}{96\eta_{B}L}{\left(1-{\bar{\delta }}_{m}+\frac{\eta_{B}vL}{∆pd^{2}}\right)}^{3}+\frac{vWd}{2}\left(1-{\bar{\delta }}_{m}+\frac{\eta_{B}vL}{∆pd^{2}}\right)-\frac{\bar{\delta }∆pWd^{3}}{8\eta_{B}L}{\left(1-{\bar{\delta }}_{m}+\frac{\eta_{B}vL}{∆pd^{2}}\right)}^{2}\;\;\;\;\;\;\;\;\;\;\;\;+\frac{∆pWd^{3}}{2\eta_{B}L}\left[\frac{1}{12}{\left(1+{\bar{\delta }}_{m}+\frac{\eta_{B}vL}{∆pd^{2}}\right)}^{3}-\frac{1}{2}{\left(1+{\bar{\delta }}_{m}+\frac{\eta_{B}vL}{∆pd^{2}}\right)}^{2}+\left(1+{\bar{\delta }}_{m}+\frac{\eta_{B}vL}{∆pd^{2}}\right)-\frac{2}{3}\right]$$

Since the flow caused by the effective area of the piston squeezing the compression chamber when the piston moves is equal to the sum of the flow $Q_{Bm}$ in the damping channel, the equation is expressed as follows [39,40]:

(28) $$Q_{Bm}=A_{p}v=Q_{Bm}$$

When ∆p≫v (under a strong magnetic field or large yield stress), Equation (28) can be simplified as:

(29) $$Q_{Bm}=-\frac{∆pWd^{3}}{96\eta_{B}L}{\left(1-{\bar{\delta }}_{m}\right)}^{3}-\frac{\bar{\delta }∆pWd^{3}}{8\eta_{B}L}{\left(1-{\bar{\delta }}_{m}\right)}^{2}+\frac{∆pWd^{3}}{2\eta_{B}L}\left[\frac{1}{12}{\left(1+{\bar{\delta }}_{m}\right)}^{3}-\frac{1}{2}{\left(1+{\bar{\delta }}_{m}\right)}^{2}+\left(1+{\bar{\delta }}_{m}\right)-\frac{2}{3}\right]$$

Substituting Equations (23) and (29) into Equation (28) to obtain the Bingham coefficient $B_{i}$ :

(30) $$Bi=\frac{1}{\frac{A_{d}}{192}\frac{{\left(1-{\bar{\delta }}_{m}\right)}^{3}}{{\bar{\delta }}_{m}}+\frac{A_{d}}{16}{\left(1-{\bar{\delta }}_{m}\right)}^{2}-\frac{A_{d}}{4{\bar{\delta }}_{m}}\left[\frac{1}{12}{\left(1+{\bar{\delta }}_{m}\right)}^{3}-\frac{1}{2}{\left(1+{\bar{\delta }}_{m}\right)}^{2}+\left(1+{\bar{\delta }}_{m}\right)-\frac{2}{3}\right]}-\frac{1}{2}$$

where $A_{d}=Wd$ is the cross-sectional area of the damping channel.

From the above analysis, for the parallel plate model, Table 4 indicates the damping coefficient of the damper produced by the Bingham constitutive fluid flowing in the damping channel for the mixed flow mode.

## 5. Theoretical Analysis of Displacement Controllable Magnetorheological Damper under Impact Environment (Take the Anti-Recoil as an Example)

**Figure 6:**
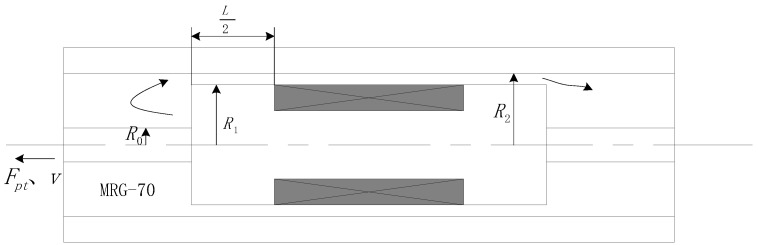
The basic structure of the damper.

For the Bingham constitutive model, the fluid working at the mixed mode is the closest to the actual working condition of the damper. Therefore, the following is a preliminary exploratory analysis of the controllability of the dampers using MRG-70 in the damping channel working at the mixed flow under different magnetic fields. The basic mechanism and dimensions of the designed magnetorheological damper are presented in Figure 6 and Table 5. Table 6 lists the Bi and $C_{Bm}$ under the magnetic field at 0 mT, 131 mT, 264 mT, 528 mT, and 1056 mT with the size presented in Table 6.

**Figure 7:**
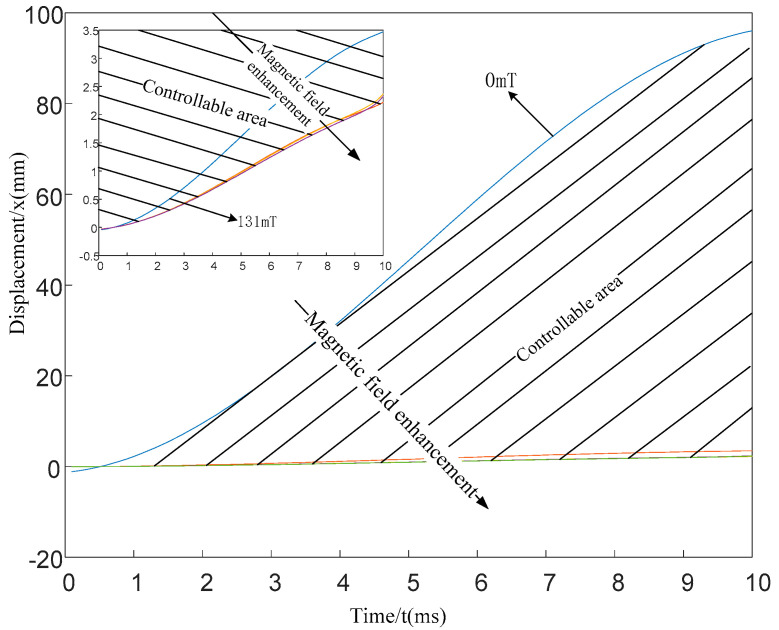
The displacement of the artillery recoil during bore period under different magnetic fields.

Figure 7 presents the displacement movement of the gun during the recoiling period when the magnetic induction intensity is 0 mT, 131 mT, 264 mT, 528 mT, and 1056 mT by setting the strength of the current flowing into the solenoid coil is 0A, 1A, 2A, 3A, and 4A, respectively (the gun’s high and low firing angle is 60°).

It can be observed from Figure 7 that when the artillery recoil is under the action of the resultant force, the recoil device is displaced, and the displacement direction is the same as the direction of the resultant force. Without the control of the magnetic field, the recoil displacement caused by the resultant force changes drastically with time, and it rapidly increases from 0 mm to 95 mm during the barrel period (about 10 ms). With the introduction of magnetic field control, when the applied magnetic induction intensity is 131 mT, the recoil only moves 3.5 mm under the force, and the controllability is 91.5 mm (defining controllability as the displacement change caused by the magnetic field). While continuing to increase the magnetic induction, the recoil displacement does not change significantly. After increasing from 131 mT to 1056 mT, the recoil displacement drops from 3.5 mm to 2.5 mm, and the controllability is 1mm. The shaded area in Figure 7 represents the controllable area. For the convenience of observation, the small window in Figure 7 presents the control curve of the damper on the recoil displacement after the magnetic induction intensity is 131 mT. The above analysis indicates the feasibility of using magnetorheological dampers to control the recoil process of the artillery, and the power consumption is very small (most of the shadow area is between the 0–131 mT curve of the magnetic induction intensity).

It is worth mentioning that in Figure 7, it is observed that without magnetic field control, at the beginning of the barrel period, the friction of the projectile against the inner wall of the barrel drives the recoil to lean forward, so the initial displacement is negative. This recoil forward effect seriously affected the accuracy of the artillery launch. After the magnetic field control is added, because MRG-70 has a shear yield stress, it can play a role in resisting forward tilt. The specific control mechanism needs to be studied in depth in the next work.

## 6. Conclusions

The MRG-70 was developed and flow characteristics were tested. Taking the recoil process of the gun as the research background, the damping characteristics of the damper were theoretically analyzed. Finally, the flow characteristic parameters of MRG-70 are substituted into the theoretical analysis results to conduct a preliminary analysis of the controllability of the magnetorheological damper applied in the anti-recoil of the artillery. The results are as follows: (1) For different magnetic fields, the constitutive characteristics of the self-developed MRG-70 can be captured by the Bingham model. (2) The damping coefficient of the damper increases along with the dynamic viscosity $\eta_{B}$ of the MRG-70, the effective working cross-sectional area of the piston $A_{p}$, the magnetic pole length L, and the Bingham coefficient Bi. However, it is decreases along with the square of the y-axis width d of the damping channel and the dimensionless width ${\bar{\delta }}_{m}$ in the y-axis direction of the plunger flow before yielding. (3) The magnetorheological damper using MRG-70 feature with controllability during the recoil period of the artillery, and can achieve a wide controllable range when the energy input is very small under the magnetic flux density ranging from 0 mT to 131 mT.

## Figures and Tables

**Table 1:** Materials was used to produce organic silicon oligomer.

Materials	Specification	Manufacturer
Dimethyldiethoxysilane (DDS)	≥98%	Sinopharm Chemical Reagent Co., Ltd. China
Monomethyltriethoxysilane (MTS)	≥98%	Sinopharm Chemical Reagent Co., Ltd. China
Diphenyldiethoxysilane	≥98%	Jiangsu Sanmu Chemical Reagent Co., Ltd. China
Phenyldiethoxysilane (PES)	≥98%	Jiangsu Sanmu Chemical Reagent Co., Ltd. China
Ethyl acetate	Industry	Nanjing Guochen Chemical Co., Ltd. China
Butanol	Industry	Nanjing Guochen Chemical Co., Ltd. China
water	Pure water	Nanjing Pure Water Company. China
Phenyltrichlorosilane	≥98%	Hebei Taifeng Chemical Co., Ltd. China

**Table 2:** Each parameter value of Bingham model at different magnetic induction intensity.

Magnetic Field/mT	0	131	264	528	1056
$\tau_{B}$/Pa	6.78	2445.66	8515.72	20,010.39	21,028.84
$\eta_{B}$/Pa·s	1.32	37.41	61.84	63.97	65.69

**Table 3:** Boundary conditions for a mixed flow model of a damper parallel plate.

Working Mode	Pressure Gradient	Boundary Conditions of Bingham Constitutive Model		
Mix mode	$\frac{\partial p}{\partial z}=\frac{∆p}{L}$	Region2 $y_{mpi}<y<y_{mpo}$	Region1 $0<y<y_{mpi}$	Region3 $y_{mpo}<y<d$
		$\{\begin{array}{c} \tau_{Bm}\left(y_{mpi}\right)=\tau_{B}+\frac{\eta v}{d}\\ \tau_{Bm}\left(y_{mpo}\right)=-\tau_{B} \end{array}$	$\{\begin{array}{c} u_{Bm}\left(0\right)=v\\ u_{Bm}^{\prime }\left(y_{mpi}\right)=0 \end{array}$	$\{\begin{array}{c} u_{Bm}\left(d\right)=0\\ u_{Bm}^{\prime }\left(y_{mpo}\right)=0 \end{array}$

**Table 4:** The damping coefficient of the damper in Bingham constitutive fluid damping channel flows under mixed flow mode.

Working Mode	Constitutive Model	Damping Coefficient	Bingham Coefficient
Mix flow	Bingham	$C_{Bm}=\frac{\eta_{B}A_{p}L\left(2Bi+1\right)}{{\bar{\delta }}_{m}d^{2}}$	$Bi=\frac{1}{\frac{A_{d}}{192}\frac{{\left(1-{\bar{\delta }}_{m}\right)}^{3}}{{\bar{\delta }}_{m}}+\frac{A_{d}}{16}{\left(1-{\bar{\delta }}_{m}\right)}^{2}-\frac{A_{d}}{4\bar{\delta_{m}}}\left[\frac{1}{12}{\left(1+{\bar{\delta }}_{m}\right)}^{3}-\frac{1}{2}{\left(1+{\bar{\delta }}_{m}\right)}^{2}+\left(1+{\bar{\delta }}_{m}\right)-\frac{2}{3}\right]}-\frac{1}{2}$

**Table 5:** The basic size of the damper.

$R_{0}$	$R_{1}$	$R_{2}$	L
5 mm	10 mm	12 mm	20 mm

**Table 6:** The two coefficients (Bi and $C_{Bm}$) along with applied magnetic field with the size of the damper listed in Table 6.

Coefficient	0 mT	131 mT	264 mT	528 mT	1056 mT
Bi	0.13	3.28	4.15	4.85	4.91
$C_{Bm}$	32.11	285.21	332.52	351.01	356.99
